# Editorial: Abiotic stress and physiological adaptive strategies of insects

**DOI:** 10.3389/fphys.2023.1210052

**Published:** 2023-05-04

**Authors:** Seema Ramniwas, Pankaj Kumar Tyagi, Aanchal Sharma, Girish Kumar

**Affiliations:** ^1^ University Center for Research and Development, Chandigarh University, Mohali, India; ^2^ Noida Institute of Engineering and Technology (NIET), Noida, India; ^3^ The Gosnell School of Life Sciences, Rochester Institute of Technology, Rochester, NY, United States

**Keywords:** abiotic stress, insect, physiology, climate change, adaptation

Abiotic stress, such as extreme temperatures, hypoxia, or nutrient deficiencies, is an inherent part of every ecosystem and can have a range of impacts on insects. These stresses can affect the behavior, development, reproduction, and survival of insect populations, potentially altering the balance of predator-prey relationships and disrupting ecosystem functions. In response to threseese stresses, insects may exhibit physiological, biochemical, or behavioral adaptations to cope with adverse conditions, but if the stress is severe or prolonged, it can lead to population declines or even extinctions. Therefore, understanding the strategies employed by insects to respond to these challenges is crucial to predict and mitigate the impact of climate change on insect populations and the ecosystems they inhabit. By gaining insight into how insects respond to diverse abiotic stresses, we can develop effective conservation strategies to protect insect populations and maintain ecosystem health.

This Research Topic features ten research articles resulting from studies conducted in four different countries (China, India, Mexico, and United States), demonstrating the significance of collaborative science. The published articles add to the expanding literature on the physiological adaptations of insects in response to diverse abiotic stresses and provides valuable insights and knowledge that can inform future research in this area and help develop effective strategies for the conservation of insect populations in the face of climate change.

Temperature is one of the most stressful abiotic pressures. Exposure to elevated temperatures have been observed to negatively affect insect growth and development, leading to reduced fecundity, longevity, and dispersal (Ramniwas and Kumar, 2019). The study investigating the fruit fly genus *Anastrepha*, including species such as *A. ludens*, *A. obliqua*, *A. striata*, and *A. serpentine*, has revealed that lifespan can vary among different species even when exposed to the same constant temperature Guillén et al. Interestingly, the study discovered that *A. obliqua*, which typically thrives in hot environments, exhibited an unexpected cold hardiness. Furthermore, the study found that thermal stress could affect the lifespan of male and female insects differently.

Even brief periods of heat exposure can significantly impact reproductive processes and fertility, as reported by Walsh et al. (2021). This assertion is reinforced by the work of Li et al. in their examination of the fruit fly *Zeugodacus tau*. Their research revealed that short-term exposure to high temperatures (34°C–44°C) for only 12 h led to changes in the mating behavior, antioxidant defence, and detoxifying enzymes of fruit flies in a sex-specific manner. These findings could have significant ecological implications for the survival, reproduction, and operational sex ratio of populations experiencing heat stress.

The impact of artificial light at night (ALAN) on biodiversity and ecosystem processes is gaining recognition as a significant threat apart from thermal stress (Sanders et al., 2021). ALAN has been suggested as a driver of insect declines (Grubisic et al., 2018; Owens et al., 2020), as it has diverse negative effects on insects throughout their life-cycles, including reduced adult activity, increased predation, and disrupted reproduction (Owens and Lewis, 2018; Boyes et al., 2021). Jiang et al. investigated the effects of ALAN on the locomotion and oviposition of *Dastarcus helophoroides*. The study revealed a decrease in the egg-laying capacity and locomotor activity of *D. helophoroides* under bright artificial light exposure (1–100 lx) at night.

Heat shock proteins (Hsps) act as molecular chaperones that respond to both biotic and abiotic stressors. Investigating the correlation between thermal acclimation and the expression of specific Hsps is crucial for gaining a deeper understanding of the molecular mechanisms involved in the heat response. Quan et al. conducted a study where they subjected *Ostrinia furnacalis*, the Asian corn borer, to temperatures of 33°C and 43°C to evaluate the expression of particular Hsps. They observed a significant upregulation of Hsp70 and Hsp90 transcripts within one to 2 h of sustained heat stress at 43°C, indicating a quick onset of these Hsps under extreme thermal stress. In a separate study, Barman et al. examined how *Bemisia tabaci*, the whitefly, expresses Hsps when exposed to different temperature conditions. The study showed that Hsp70 expression was induced by both cold (12°C) and hot (44°C) temperatures, suggesting its role in adapting to both heat and cold. Moreover, Hsp40 transcript levels were significantly upregulated under extreme temperature conditions, with a higher expression at 44°C, indicating a possible role in heat adaptation for *B. tabaci*.

Several studies have found a positive association between higher levels of Hsps and an increase in thermotolerance in organisms. However, Bowler (2005) has noted that the expression of Hsps varies between populations acclimated to different thermal histories. King and Stillman conducted a study on larval caddisflies (*Dicosmoecus gilvipes*) from three different eco-regions (mountain, valley, and coast) and exposed them to current and future summertime temperatures. The results indicated that there were population-specific patterns of gene expression in response to controlled and daily warming conditions, which suggests that local acclimatization or adaptation may differentiate populations. Nevertheless, the similarity in responses to extreme temperatures across populations indicates that the response to thermal stress is constrained or channeled, as highlighted in King and Stillman’s study.

Despite the potential for adverse effects such as environmental contamination, insecticide resistance, and threats to human health, the primary method for managing insect pests is the use of synthetic chemical insecticides. Insects adapt to insecticides by modulating their gene expression, emphasizing the need to investigate the molecular basis of this adaptation to develop alternative control methods. Li et al. explored the expression of five chitinase-like proteins (ApIDGF, ApCht3, ApCht7, ApCht10, and ApENGase) in pea aphids (*Acyrthosiphon pisum*) exposed to various abiotic stresses, including temperature, insecticides, and 20-hydroxyecdysone (20E) stress. The study found that the expression of these five genes was differentially regulated by different stresses. For instance, ApCht7 expression was upregulated at low temperatures (10°C), while insecticide exposure (imidacloprid) downregulated the expression of all five genes. These findings provide insight into the role of chitinase-like proteins in abiotic stress management and can be beneficial in managing pea aphids under multiple stresses. In another study, Khalid et al. investigated the effect of cyromazine, a bio-rational insecticide, on the germ cells of *Drosophila*. The study suggested that cyromazine impacts the ecdysone signaling pathway, leading to a decrease in the number of germ cells. This highlights the ability of chemical insecticides to interfere with the biochemical and reproductive pathways of insects. In an effort to replace or decrease the use of chemical insecticides, recent research has focused on developing alternative control methods. Asad et al. examined the efficacy of a CRISPR/Cas9-mediated gene-drive construct for *Plutella xylostella*, a highly-destructive lepidopteran pest. The genetically modified construct had high gene-drive efficiency and could transmit desired traits to the pest’s offspring, effectively controlling pests of cruciferous crops. The study suggests that it is possible to develop highly effective gene-edited constructs for other destructive pest species related to *P. xylostella*.

Fish often encounter hypoxia, a condition of insufficient oxygen in the cell, which can have adverse effects on their survival. To cope with this challenge, fishes have developed versatile mechanisms to acclimate to oxygen deficiency in their habitats (Mandic et al., 2009). Chang et al. investigated the molecular mechanism underlying the acclimation response to hypoxia in *Litopenaeus vannamei*, which is one of the most widely cultivated shrimp species worldwide. Their study used quantitative real-time PCR analysis and revealed differential expression of hemocyanin, chitinase, heat shock protein 90 (Hsp90), programmed death protein, and glycogen Phosphorylase, suggesting their role in hypoxia acclimation. The findings of this study can enhance the overall understanding of hypoxic stress in *L. vannamei* and the identified differentially expressed proteins could be utilized to support breeding programs for developing new strains of *L. vannamei* with enhanced tolerance to hypoxia.

In conclusion, Abiotic factors like temperature and artificial light impact insects’ physiology and behavior, including survival, reproduction, and fitness. They can trigger stress responses, including heat shock protein expression, which varies across populations and species. Insects have developed resistance to chemical insecticides by modulating gene expression. Understanding the molecular mechanisms behind insect responses to stress can aid in developing sustainable pest control methods. More research is needed to comprehend the ecological implications of abiotic stressors on insect populations.
